# Current Therapies for Neonatal Hypoxic–Ischaemic and Infection-Sensitised Hypoxic–Ischaemic Brain Damage

**DOI:** 10.3389/fnsyn.2021.709301

**Published:** 2021-08-24

**Authors:** Konstantina Tetorou, Claudia Sisa, Arzo Iqbal, Kim Dhillon, Mariya Hristova

**Affiliations:** Perinatal Brain Repair Group, Department of Maternal and Fetal Medicine, UCL Institute for Women’s Health, London, United Kingdom

**Keywords:** hypoxia, ischaemia, neonatal encephalopathy, infection, neonatal brain damage

## Abstract

Neonatal hypoxic–ischaemic brain damage is a leading cause of child mortality and morbidity, including cerebral palsy, epilepsy, and cognitive disabilities. The majority of neonatal hypoxic–ischaemic cases arise as a result of impaired cerebral perfusion to the foetus attributed to uterine, placental, or umbilical cord compromise prior to or during delivery. Bacterial infection is a factor contributing to the damage and is recorded in more than half of preterm births. Exposure to infection exacerbates neuronal hypoxic–ischaemic damage thus leading to a phenomenon called infection-sensitised hypoxic–ischaemic brain injury. Models of neonatal hypoxia–ischaemia (HI) have been developed in different animals. Both human and animal studies show that the developmental stage and the severity of the HI insult affect the selective regional vulnerability of the brain to damage, as well as the subsequent clinical manifestations. Therapeutic hypothermia (TH) is the only clinically approved treatment for neonatal HI. However, the number of HI infants needed to treat with TH for one to be saved from death or disability at age of 18–22 months, is approximately 6–7, which highlights the need for additional or alternative treatments to replace TH or increase its efficiency. In this review we discuss the mechanisms of HI injury to the immature brain and the new experimental treatments studied for neonatal HI and infection-sensitised neonatal HI.

## Introduction

The interruption of blood and oxygen supply to the foetal brain during pregnancy and at the time of birth is a leading cause of neonatal hypoxic–ischaemic (HI) brain damage. Also known as neonatal hypoxic–ischaemic encephalopathy (HIE), this condition affects 1–3 per 1000 live births in developed countries, increasing to 26 per 1000 in the developing world (Rocha-Ferreira and Hristova, 2016). Despite the advantages in neonatal health care, a quarter of all neonatal deaths is due to HIE (Lawn et al., 2005; Rocha-Ferreira and Hristova, 2016), and 30% of the sufferers of neonatal HI brain damage develop disabilities, including cerebral palsy, seizures, and cognitive and memory impairment (Rocha-Ferreira and Hristova, 2016; Lundgren et al., 2018).

**FIGURE 1:**
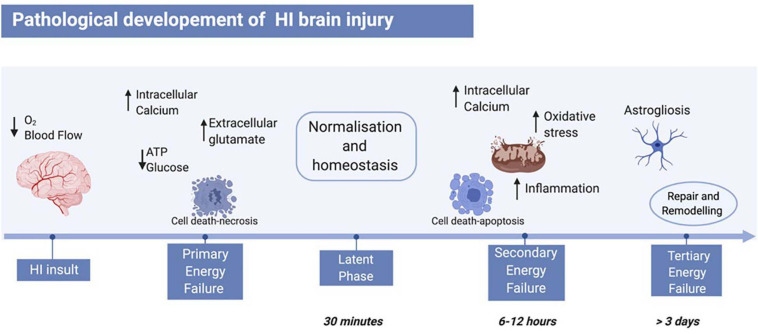
Pathological development of neonatal HI brain injury. The HI insult is initiated by reduction of blood flow and oxygen to the foetal brain, leading to primary energy failure. The main events of this phase include reduction of ATP and glucose, increase of intracellular calcium, and therefore increase of extracellular glutamate. This leads to cell death mainly *via* necrosis. Following re-oxygenation, a latent phase begins, where the body resumes a “normal” state. A secondary energy failure may take place after 6–12 h post-HI insult, where a subsequent and stronger wave of cell death hits the brain, and events like inflammation, oxidative stress, and mitochondrial damage occur. Depending on the severity of the insult, a tertiary energy failure can occur and persist for months, characterised by brain remodelling and repair, as well as astrogliosis. Figure created with BioRender.com.

The pathology of HI brain injury evolves over days *via* three consecutive phases (primary, secondary, and tertiary energy failure, Figure 1; Sarnat and Sarnat, 1976). Immediately after the HI insult, the lack of oxygen and glucose reduces mitochondrial phosphorylation and adenosine triphosphate (ATP) availability causing anaerobic respiration (Vannucci, 1990; Jensen et al., 1999). The change in metabolism results in extracellular acidosis leading to ionic pumps dysfunction, thus increasing the intracellular calcium influx, and changing the membrane potential. The depolarised neuronal membrane releases high concentrations of glutamate, which are typically cleared *via* the glia reuptake pumps during aerobic respiration, establishing an excito-oxidative cascade (Rocha-Ferreira and Hristova, 2016) causing neurotoxicity (Sanders et al., 2010) and mostly necrotic cell death (Rocha-Ferreira and Hristova, 2016). After successful re-oxygenation, a latent recovery phase takes place, where respiration switches back to aerobic and homoeostasis is recovered (Vannucci, 1990; Jensen and Berger, 1991; Gunn et al., 1992; Jensen et al., 1999). Depending on the severity of the HI insult, primary energy failure might not be compensated and would lead to secondary energy failure (Rocha-Ferreira and Hristova, 2016). This phase starts as early as 6–12 h after the initial injury and involves continued excitotoxicity, mitochondrial impairment, and inflammation. In particular, there is an increased expression of pro-inflammatory cytokines, such as interleukin-1α (IL-1α), interleukin-6 (IL-6), and tumour necrosis factor-α (TNF-α) which enhances free radical formation and cell death. Oligodendrocyte progenitors supply energy to myelinated axons and have high metabolic demand. Therefore, they are particularly sensitive to free radical formation (Janowska and Sypecka, 2018). Hence, following HI, oligodendrocyte degeneration and hypomyelination are enhanced in animal models, as well as in human newborns (Segovia et al., 2008; Janowska and Sypecka, 2018). The mitochondrial dysfunction following HI insult boosts oxidative stress by upregulating catalase (CAT), superoxide dismutase (SOD), and glutathione peroxidase (GPx), and by increasing glutathione peroxidase/creatinine ratio (GPx/Cr) (Hope et al., 1984; Penrice et al., 1997) thus causing generation of reactive oxygen species (ROS). The majority of cell death occurs *via* necrosis, apoptosis [caspase 3 dependent, Bcl-2-associated X protein (Bax)/B-cell lymphoma 2 (Bcl-2) pathway], autophagy, and apoptosis–necrosis continuum leading to cellular atrophy (Peng and Greenamyre, 1998; Puka-Sundvall et al., 2000; Johnston et al., 2002; Northington et al., 2007). Depending on the length and the severity of the HI insult, tertiary energy failure can occur and persist for weeks and months, involving remodelling and repair, astrogliosis, and late cell death (Rocha-Ferreira and Hristova, 2016).

Several studies highlight the latent period as the therapeutic window in neonatal HI because, although magnetic resonance imaging (MRI) and histological assessments show no obvious changes, cell death pathways are still active and lead to secondary and eventually tertiary energy failures. Hence, during the latent period, the pathogenesis of the disease can be interrupted and the brain damage contained by fighting the onset of the secondary energy failure (Gunn, 2000; Gunn and Thoresen, 2006; Shankaran, 2009).

In the majority of HI cases, multiple factors contribute to the damage. The presence of bacterial infection which increases the risk of intraventricular haemorrhage and brain damage (Dammann and Leviton, 2008) is recorded in 50% of preterm births (Suff et al., 2016). The exposure of the immature brain to an inflammatory stimulus causes an increase in pro-inflammatory cytokine levels and neuronal death thus leading to impairment of the natural development of the CNS (Hagberg et al., 2015). Elevated levels of pro-inflammatory cytokines such as IL-1α, IL-6, IL-8, and TNF-α in the cerebrospinal fluid (CSF) and blood serum of neonates with HI sensitise the immature brain to injury and increase the risk of development of cerebral palsy and other disabilities (Sävman et al., 1998; Foster-Barber et al., 2001; Hagberg et al., 2015; Martinello et al., 2019b).

**FIGURE 2:**
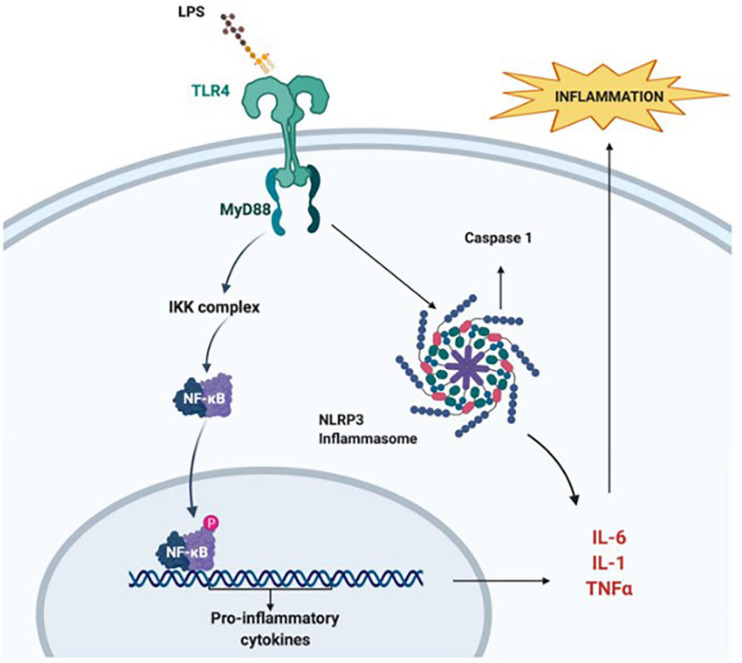
Lipopolysaccharide sensitisation. A bacterial infection sensitises the brain to HI insult *via* the interaction of LPS and TLR4. This leads to internalisation of NF-κB, mediated by MyD88. NF-κB activates the transcription of pro-inflammatory cytokine genes. Simultaneously, the interaction of LPS with TLR4 activates the NLRP3 inflammasome, which also promotes increase in pro-inflammatory cytokine levels and apoptosis. Figure created with BioRender.com.

Bacterial lipopolysaccharide (LPS) is the major component of the outer membrane of most Gram-negative bacteria and has strong immune-stimulatory proprieties (Wang et al., 2009). In rodent studies pre-exposure to LPS enhances tissue damage, mortality rate, and infarction volume following HI (Wang et al., 2009; Rocha-Ferreira et al., 2015). In the LPS-sensitised HI brain, the interaction between LPS and Toll-like receptors (TLR) appears to be critical (Lehnardt et al., 2003). The activation of TLR3 and TLR4 reduces myelination while increasing glial activation (Hagberg et al., 2015), BBB impairments, and infiltration of peripheral immune cells (Stolp et al., 2007). Accordingly, in LPS-sensitised HI, monocyte chemoattractant protein-1 (MCP-1), and cytokine-induced neutrophil chemoattractant-1 (CINC-1) expression increases to recruit peripheral monocytes (Brochu et al., 2011). Evidence suggests that TLR4 mediates the LPS-sensitisation, *via* direct binding to the receptor and activation of the myeloid differentiation factor-88 (MyD88) pathway which leads to an increase in NF-κB and TNF-α levels (Lehnardt et al., 2002; Mallard Anders Elmgren et al., 2009; Wang et al., 2009) as shown in Figure 2. Studies using TNF cluster knock-out mice (Kendall et al., 2011), MyD88 deficient mice (Mallard Anders Elmgren et al., 2009), or pharmacological inhibition of NF-κB (Yang et al., 2013a) show a reduction in brain injury after LPS-sensitised neonatal HI. The nuclear translocation of NF-κB leads to pro-inflammatory cytokines gene expression, and the activation of the inflammasome *NLRP3*, which is a caspase 1 and IL-1α activating multi-protein complex (Cunha et al., 2016; Serdar et al., 2019). However, early-onset sepsis in term babies is also caused by Gram-positive bacterial species in more than 90% of the cases, thus sensitising the neonatal brain to HI injury. The neuroinflammatory response triggered through the Gram-negative route (TLR4) is different from the one induced through the Gram-positive route (TLR2) (Falck et al., 2017). Peptidoglycans and lipoteichoic acid on the wall of Gram-positive bacteria bind to TLR2 and induce inflammatory activation *via* a different pathway, which similarly to TLR4 causes an increase of MyD88, and NF-κB and TNF-α, respectively (Takeuchi et al., 1999; Oliveira-Nascimento et al., 2012) thus exacerbating HI-induced neuronal tissue loss and demyelination in neonatal mice (Mottahedin et al., 2017).

Microglia are the primary CNS immunocompetent cells and play a central role in normal and LPS-sensitised HI. Neonatal HI induces early pro-inflammatory microglial (M1) activation. This triggers synthesis and secretion of pro-inflammatory cytokines, such as IL-1 and TNF-α, thus promoting inflammation and exacerbating damage. On the other hand, M2 activated microglial cells produce anti-inflammatory cytokines like IL-4 and IL-10, and in communication with other cells mediate anti-inflammatory immune response and promote healing (Mantovani et al., 2004).

In a rat model of infection-sensitised HI, microglial cells display a pro-inflammatory M1 phenotype at 24 h post-insult (Serdar et al., 2019). At the same time, the expression of genes corresponding to an anti-inflammatory M2 microglial phenotype was also recorded (Serdar et al., 2019) highlighting that microglia play a dual role in normal and LPS-sensitised HI and can switch between pro- and anti-inflammatory phenotype while at times simultaneously expressing both M1 and M2 markers.

## Animal Models of Hypoxia–Ischaemia

This review aims to provide an update on the new proposed treatments which are studied for neonatal HI and infection-sensitised HI. To better understand this, we offer a summary of the literature around the animal models used for these two kinds of neonatal HI.

### Rodents

Most studies investigating neonatal HI focus on using rodent models with the most prevalent and best studied of these being the one developed by Rice and Vannucci (Rice et al., 1981; Rumajogee et al., 2016; Millar et al., 2017). In brief, the Rice–Vannucci model involves unilateral ligation of the common carotid artery, followed by exposure to 8–10% oxygen for 30 min to 3 h at 37°C. Injury is restricted to the ipsilateral hemisphere, thus allowing the contralateral hemisphere to be used as a control. The Rice–Vannucci model produces an injury profile similar to the human foetal brain, with cortex, subcortical and periventricular white matter, striatum, thalamus, and hippocampus being the most damaged regions due to their high metabolic requirements (Rice et al., 1981; Martin et al., 1997; Johnston et al., 2001; McQuillen et al., 2003; Vannucci and Hagberg, 2004). Initially developed in the rat, this model has also been successfully modified and extended to the mouse (Sheldon et al., 1998).

One of the main advantages of this model is its flexibility in replicating both preterm (rodent postnatal days 1–6) and term (rodent postnatal days 7–10) human foetal injury (Jisa et al., 2018).

On the other hand, a significant limitation of the Rice–Vannucci model is the unilateral nature of the insult, inducing focal brain injury, which is not fully representative of the clinical observations and leading to considerable between-animal variability in the degree of damage, ranging from mild to severe (Vannucci and Hagberg, 2004). Moreover, there is variance in the damage profile between different mouse strains which raises a reproducibility issue (Sheldon et al., 1998; Rocha-Ferreira et al., 2015; Ann Sheldon et al., 2019).

Rodent models of bilateral carotid artery occlusion have also been developed (Uehara et al., 1999; Cai et al., 2001), involving postnatal day 1 or 5 rats without hypoxic conditions. The neuropathological observations at 48 h post-surgery indicate mild to severe white matter lesions in the internal capsule and cerebral cortex as well as a 25% reduction in CSF volume. Subsequent rodent models include bilateral carotid artery occlusion in postnatal day 4 rats combined with 10–15 min exposure to 8% oxygen, which causes mild to severe injury with reduced numbers of mature oligodendrocytes, impaired myelination, and compromised behavioural response including locomotor activity and memory deficits (Fan et al., 2005). A rat model of bilateral common carotid artery occlusion with temporary ligation has been developed in postnatal day 7 pups (Jelinski et al., 1999) where both arteries were ligated for 10 min while the animals were simultaneously exposed to 8% oxygen. The resulting injury was characterised with fewer oligodendrocytes both 6 and 24 h post-HI with no changes in astrocyte numbers. Despite better reflecting HI injury in humans, these bilateral models, have limited use due to their high mortality rate.

In addition to the postnatal rodent HI models, prenatal ones have also been developed. In those HI is induced in rats at E17 by clamping the uterine vasculature for 30 min. As a result, foetal brain iNOS activity is increased (Cai et al., 1998), and NMDA receptor expression is altered (Cai and Rhodes, 2001).

In addition to mice and rats, guinea pigs have also been used to model HI. Their longer gestation and similarity with the human pattern of prenatal brain development make them an ideal rodent *in utero* HI model (Hirst et al., 2018). Unilateral uterine artery ligation at 30 days gestation models pre-term injury and leads to a reduced number of neurons in hippocampus and cerebellum, as well as impaired dendritic and axonal growth (Mallard et al., 2000). In a different hypoxia-only model, guinea pigs at 65 days of gestation (term injury) were exposed to 10.5% oxygen for 14 days (Dong et al., 2011). The brains of the injured animals had increased iNOS activity with inducible macrophage-type nitric oxide synthase upregulated in cerebral cortex, hippocampus, thalamus, and hypothalamus, including white and grey matter.

### Large Animal Models

Neuroanatomically, the rodent brain significantly differs from the human in both size and level of cortical gyrification. This limitation can be overcome by the use of large animal models with gyrencephalic brains more similar in size to the human ones.

Non-human primate models of HI have been developed in several species including rhesus monkeys (Ranck and Windle, 1959; Faro and Windle, 1969), in which detaching of the placenta through hysterotomy near term causes total asphyxiation. As a result, the subsequent damage is consistent with the one observed in humans and predominantly localised to the brainstem including sensory and motor nuclei as well as the basal ganglia, affecting both white and grey matter. However, differently from humans, little change is seen in the hippocampus. Further studies developed this model in the *Macaca nemestrina* monkeys in which the umbilical cord of near term foetuses is clamped for 12–15 min followed by delivery *via* hysterotomy (Juul et al., 2007). The experimental animals display gliosis and behavioural deficits such as seizures. A preterm non-primate model has also been established in baboons delivered through hysterectomy at 125 days gestation (Inder et al., 2005). In this case, the injury predominantly affects the white matter. While non-human primates are developmentally most similar to humans and provide a better basis to study long-term behavioural changes, the ethical issues and high experimental costs restrict their use in HI research (Painter, 1995).

Foetal sheep models of HI are well studied amongst the large animal models and have provided a unique insight into the pathophysiology of HI. Intermittent umbilical cord occlusion for 1 min every 2 min over a cycle of 2 h in the sheep is used to replicate uterine contractions and produces a term injury similar to HI in humans (Clapp et al., 1988; De Haan et al., 1997), with damage primarily confined to white matter. Another term model involves common uterine artery occlusion for 30–60 min, alone or combined with maternal hypoxia for 120 min, leading to hypercarbia, acidosis, and initial hypertension (Williams et al., 1992), and resulting in cortical damage.

Sheep are advantageous models for the study of HI due to the larger brain and the neurodevelopmental similarity with the human foetus, however, the higher experimental costs restrict their use (Back et al., 2012).

Piglet models of HI are also well-studied thanks to the developmental and neuroanatomical similarities between the human and piglet neonatal brain (Koehler et al., 2018). Severe hypoxia, without ischaemia, is induced by performing a tracheostomy and mechanically ventilating the piglet with 6% oxygen (Thoresen et al., 1996), thus causing injury mainly to the cerebral cortex, subcortical white matter and hippocampus. HI models have also been produced through bilateral carotid occlusion paired with hypoxia in newborn piglets (Edwards et al., 1995; Robertson et al., 2013) or through a combination of ischaemia with complete asphyxiation in 1-week-old piglets (Brambrink et al., 1999). Both models represent HI at term and produce damage in the parasagittal cortex, striatum, thalamus, and hippocampus.

Other known large animal HI models include a preterm rabbit model and a more recently developed ferret model. In the first one, preterm rabbit foetuses are exposed to global hypoxia through *in utero* ischaemia. As a result, the animals display hypertonia and motor control impairments resembling motor disturbances seen in humans (Derrick et al., 2004, 2007). The ferret is born lissencephalic but postnatally develops gyrencephaly with a white-to-grey ratio similar to the human (Empie et al., 2015; Falck et al., 2018; Schwerin et al., 2018). Thus, the ferret model is a promising one, because despite its smaller size, the ferret brain is structurally more similar to the human one compared to the rodent.

## Animal Models of Infection-Sensitised Hypoxia–Ischaemia

Intrauterine infection increases the vulnerability of the neonatal brain to HI injury and amplifies the risk of death and disability compared to HI alone (Grether and Nelson, 1997; Wu et al., 2003; O’Callaghan et al., 2011). Eklind et al. (2001) developed the first infection-sensitised model in 2001 with a modification of the classic Rice–Vannucci model; a single dose of LPS was administered to 7-day old rat pups 4 h before unilateral carotid artery occlusion. The LPS administration induces a more severe injury profile compared to HI alone, with larger areas of infarction and higher microglial and astroglial activation (Yang et al., 2005; Wang et al., 2009; Bonestroo et al., 2015a). The model has been successfully extended to the mouse, with LPS being administered 6–12 h prior to the HI insult (Kendall et al., 2011). Like in the Rice–Vannucci HI model, the level of severity caused by the LPS-sensitised HI depends on the mouse strain (Rocha-Ferreira et al., 2015). Similarly to LPS, Gram-positive bacterial infection sensitisation also contributes to neonatal HI injury (Falck et al., 2017). In this case, postnatal day 7 rats are intraperitoneally administered with a TLR-2 agonist [*N*-palmitoyl-*S*-(2,3-bis(palmitoyloxy)-(2R,S-propyl)-R-cysteinyl-seryl-(lysyl)-3-lysine, PAM_3_CSK_4_], 8 h prior to HI insult (Falck et al., 2017). This causes a significant increase in brain damage compared to the vehicle treated animals resulting in decreased neuronal cell count and increased hippocampal area loss.

A novel large animal model of Gram-negative infection sensitised hypoxia has been developed in the newborn piglet (Martinello et al., 2019b). A single dose of LPS administered 4 h prior to hypoxia increased mortality and exacerbated brain injury compared to hypoxia alone, with an increase in microglial and astroglial activation. This model only investigated hypoxia without ischaemic insult, thus limiting its application.

More recently, a ferret model of LPS sensitised HI brain jury has been developed, where postnatal day 17 ferrets receive an intraperitoneal injection of LPS 4 h before hypoxia. This models a late preterm human insult (Wood et al., 2019). The injured ferrets display variable degrees of damage in the cortical gyri and associated sulci, as well as behavioural deficits.

However, the sensitisation effect of LPS in HI animal models depends to a great extent on the dose and time of LPS pre-treatment. In a neonatal rat HI model, injection of 0.3 mg/kg of LPS 24 h prior to HI greatly increased microglial and macrophage activation and upregulated TNF-α and iNOS expression at 12 h post treatment, causing high HI mortality. Conversely, 0.05 mg/kg of LPS elicited very low expression of the same markers resulting in low mortality, as well as significantly better learning and memory performance, and reduced brain damage in adulthood (Lin et al., 2009). Administration of 0.01 mg/kg LPS at E15 in C57BL/6 mice exacerbated brain injury after HI at P5 and P9, whereas in adult mice (P70) LPS treatment reduced tissue loss (Wang et al., 2007a). A low dose LPS administration in foetal sheep induced specific TLRs with potential neuroprotective role after acute ischaemia (Dhillon et al., 2015). Specifically, low LPS dose administered over 5 days with the last treatment at 24 h prior to cerebral ischaemia at E94–95 attenuated inflammation and astroglial activation, and reduced apoptosis. This preconditioning effect was associated with upregulation of mRNA for TLR4, TLR7, and IFN-β, as well as a considerable increase in plasma IFN-β levels, suggesting IFN-β as an important mediator of endogenous neuroprotection (Dhillon et al., 2015). The time of LPS pre-treatment is also crucial for the effect on HI brain damage. Kendall et al. (2011) demonstrated that 0.03 mg/kg LPS injection at the time of or 24 h before HI had no significant effect on the level of brain injury in C57/Bl6 P7 mice, however, the same dose administered at 4 or 12 h prior to the insult was detrimental. Additionally, the data from Kumral et al. (2012) revealed that 24 h pre-HI treatment with a low dose of LPS significantly reduced apoptotic cell death and hypomyelination, thus suggesting neuroprotection. The choice of endotoxin for the pre-treatment is also of great importance for the outcome of the infection-sensitised HI model. For example, administration of lipoteichoic acid as a major immunogen from Gram-positive bacteria which, when bound to its target interacts with circulating antibodies and activates the complement cascade, 3 h prior to HI reduces brain injury (Hagberg et al., 2002). This suggests a high complexity of infection sensitised HI injury that needs to be taken into account when choosing an animal model.

## Differences Between Preterm and Term HI

The severity of the injury developed after neonatal HI, is highly dependent on the timing of the damage in respect of gestation. Preterm and term animal models are in fact used to investigate different aspects of HI brain injury.

In preterm infants (<32 weeks of gestation) HI generally has a more complex temporal profile, with chronic nature (Laptook, 2016; Ohshima et al., 2016). It is characterised by cognitive and sensory deficits (McQuillen et al., 2003), and the immature immune system, potentially promotes an excessive and sustained inflammatory response (Gilles et al., 2018).

At this stage, the periventricular white matter is highly susceptible and particularly struck by the insult resulting in periventricular leukomalacia (PVL) (Volpe, 2001; Johnston et al., 2002). Pre-oligodendrocyte development is hindered, thus leading to abnormal myelination typically seen in MRI scans (Back et al., 2007; Volpe et al., 2011). Pre-oligodendrocytes are in fact highly susceptible to the pro-inflammatory state and oxidative stress generated after the HI insult resulting in a large amount of cell death (Fern and Möller, 2000; Baud et al., 2004; Back et al., 2007; Segovia et al., 2008; Volpe et al., 2011). Preterm neurons are also highly vulnerable to the HI insult, as the NMDA receptors are physiologically upregulated and more permeable to calcium (Jantzie et al., 2013), making these cells susceptible to the excito-toxicity cascade.

In term infants (>36 gestational age) HI insult causes selective damage to the sensorimotor cortex, basal ganglia, thalamus (Martin et al., 1997), and brainstem (Johnston et al., 2001), resulting in severe motor disability, including rigidity, impairment of mostly the upper limbs, and speech difficulties (Menkes and Curran, 1994; Johnston et al., 2001). Cerebral white matter is also described as selectively sensitive to term HI injury, with abnormalities of watershed white matter and cortex present in 40–60% of patients (Huang and Castillo, 2008).

The changes in NMDA receptor expression during neurodevelopment could explain the different patterns of injury seen in the preterm versus term infants. A rat HI model using intracerebral injection of glutamate receptor agonist caused selective white matter injury at P7 (modelling preterm) compared to severe cortical infarction with no white matter susceptibility at P10 (term) (McLean and Ferriero, 2004).

## Current Treatments

### Therapeutic Hypothermia

Therapeutic hypothermia (TH) is a clinical procedure where a patient temperature is lowered from 36 to 33.5°C, aiming to counteract an event of energy drop by reducing cell metabolism and energy requirements (Sisa et al., 2017). In neonatal HI brain damage TH is the standard treatment applied in moderate to severe injury through selective head or whole body cooling, showing satisfactory results in 11 clinical trials. TH reduces the possibility to develop cognitive impairments and disabilities (Gluckman et al., 2005; Jacobs et al., 2007; Srinivasakumar et al., 2013).

Despite the promising results, TH does not guarantee total recovery from neonatal HI and 40% of treated infants still develop disabilities (Ezzati et al., 2016). Obvious limitations of TH associate with immunosuppression, slow drug metabolism and clearance, and the increase of energy expenditure through the physiological activation of thermoregulatory mechanisms (Sisa et al., 2017).

Rat and piglet models of LPS-sensitised HI report increased mortality rate and tissue damage, no matter whether the neonates underwent treatment with TH or not (Osredkar et al., 2014, 2015). Similarly, clinical studies on neonates exposed to intrauterine infection, report that TH does not result in neuroprotection (Wintermark et al., 2010). Overall, such findings suggest that despite that TH is the current standard treatment for neonatal HI brain damage, it is not protective in LPS-sensitised HI cases. Importantly, preclinical models of infection sensitisation suggest TH to cause even more damage to the injured brain (Martinello et al., 2019a).

The mechanism by which LPS-induced sensitisation overcomes the neuroprotective effects of TH is still unknown. A possible explanation relies on the inter-individual variability, as suggested by a study where the damage from HI alone or combined with pre-exposure to LPS were investigated in different mouse strains (Rocha-Ferreira et al., 2015). As a result, the genotype seemed to play a critical role in the individual response to both infection-sensitised and HI injury alone (Rocha-Ferreira et al., 2015). In addition, clinical studies in neonates who underwent TH treatment after HI alone suggest body cooling to be immunosuppressive (Nakamura et al., 2013; Chalak et al., 2014), through a reduction of the number of circulating leucocytes and chemokines (Jenkins et al., 2013). Therefore, TH might be counteracting the physiological attempt of the immune system in fighting the bacterial infection.

As previously mentioned, Gram-positive bacterial sensitisation is also quite common, especially in the developing world (Fjalstad et al., 2015). Falck et al. (2017) reported that TH induced recovery in 80% of HI rats with Gram-positive sensitisation, suggesting that the neuroprotective effects of TH might be pathogen dependent. In line with these preclinical data, a retrospective clinical study reports encouraging outcomes with TH treatment in neonates following Gram-positive sensitised HI (Hakobyan et al., 2019).

While these recent results give hope for the use of TH in some cases of bacteria sensitised HI, this treatment still needs further exploration. Importantly, the fact that TH is only partially effective and completely ineffective in Gram-negative sensitised HI highlights the need for alternative therapeutic approaches for neonatal HI alone and combined with infection.

## Experimental HI Treatments

### Cannabinoids

The endocannabinoid system (ECS) exerts a substantial neuromodulatory role in many brain regions and is crucial for the regulation of neuronal activity (Soltesz et al., 2015). Cannabinoids, such as cannabidiol and *N*-arachidonoyl-dopamine (NADA) have emerged as promising substances ameliorating HI brain damage in neonates (Martínez-Orgado et al., 2007). There are two cannabinoid receptors; CB1 receptors are expressed in the CNS but can also be found in peripheral tissues. CB2 receptors are expressed mostly in mid- and hindbrain and less in forebrain neurons. CB2 receptors have also been observed in activated glia (Johnston et al., 2001). Cannabinoids bind to their receptors and provide neuroprotective effects through reduction of glutamate release and nitric oxide (NO) production, prevention of intracellular calcium influx, modulation of inflammation and cytokine release while protecting glial cells (Martínez-Orgado et al., 2007; Pacher and Mechoulam, 2011). CB1–CB2 agonist WIN 55122 was administrated subcutaneously in a rat model of HI and provided neuroprotection by reducing brain tissue atrophy, glial and vasogenic oedema, and by increasing cortical cells density as demonstrated through histological and MRI assessments (Fernández-López et al., 2007). Cannabidiol (CBD), the major non-psychoactive constituent of *Cannabis sativa* does not bind specifically to CB1 and CB2 receptors, but modulates several non-cannabinoid receptors and ion channels, such as GABA-A and TRPV1 receptors (Pertwee, 2004; Mechoulam et al., 2007). CBD demonstrates a broad spectrum of anti-inflammatory and anti-oxidant properties in numerous pathological conditions including ischaemic stroke and neonatal HI through inhibition of NF-κB activation and iNOS expression (Hayakawa et al., 2010). Pazos et al. (2013) report that CBD leads to long-term neuroprotection after a neonatal HI insult at P7–P10 in Wistar rats. Specifically, subcutaneous injections of CBD immediately after the HI insult resulted in a sustained neuroprotective effect associated with modulation of excitotoxicity, oxidative stress and inflammation, that persisted at 30 days after HI, with CBD-treated animals having smaller lesions and improved neurobehavioural performance when compared with the non-treated controls. Additionally, subcutaneous CBD administration 15 min or 1, 3, 6, 12, and 18 h after HI insult in mice reduced astroglial activation and tissue loss (Mohammed et al., 2017). This time point is broader than the ones reported for other neuroprotective treatments including TH. Similar histological results of reduced astroglial activation and tissue loss were observed in a piglet model of HI, where CBD also improved EEG brain activity. In this study, decrease of oxidative stress and excitotoxicity has been reported after CBD administration, through reduction of glutathione/creatine (GSH/Cr) ratio and downregulated levels of IL-1 in lesioned animals (Osredkar et al., 2014). CBD administration also has beneficial effects on remote inflammatory lung injury following cerebral HI insult in newborn pigs, by reducing leucocyte infiltration and IL-1 concentration in lung tissue (Arruza et al., 2017). Activation of serotonin 5-HT1A receptors was involved in the CBD beneficial effects on the lungs, since 5-HT1A antagonism reversed the positive outcome of CBD treatment in functional, histological, and biochemical studies.

However, in a piglet HI model high-dose cannabidiol treatment can induce significant hypotension (Garberg et al., 2017). Garberg et al. (2017) demonstrated that cannabidiol alone did not provide neuroprotective effect in a piglet HI model as indicated by neuropathology score and neurotrophic markers. They showed that cannabidiol is not neuroprotective against HI and further studies should be performed in preclinical models to confirm its safety and efficacy for subsequent tests in clinical trials (Garberg et al., 2017). Overall, cannabinoids administration after HI insult provides neuroprotection, however, the data obtained in animal models is controversial and their application in neonatal HI requires further studies.

### Quercetin

Quercetin (3,5,7,30,40-pentahydroxyflavone) is a plant flavonoid present in many plant-based foods, such as red wine, onions, green tea, and berries. It is known as health care product due to its antioxidant, anti-inflammatory and free radical scavenger properties (Erden Inal and Kahraman, 2000; McAnulty et al., 2008; Hwang et al., 2009; Qu et al., 2014).

Quercetin exerts neuroprotective effects including reduction of cortical cell apoptosis, decrease of astroglial and microglial activation and down-regulation of IL-6, IL-1β, and TNF-α in HI injured newborn rats, possibly through suppression of the TLR4-mediated NF-κB pathway (Wu et al., 2019). In addition, quercetin treatment can improve memory and spatial learning ability as well as cognitive ability in neonatal rats with white matter HI damage (Huang et al., 2012). Similar behavioural results were confirmed by Qu et al. (2014), who also showed enhancement of oligodendrocytes and oligodendrocyte progenitor cell proliferation combined with increased re-myelination after quercetin injection. *In vitro* quercetin treatment of hippocampal cell cultures subjected to ischaemic conditions prevented cell death through inhibition of excessive ROS formation and neutralisation of the irreversible cytosolic Ca^2+^ concentration increase in GABAergic neurons. Additionally, 24 h incubation with quercetin further improved neuroprotection through increased expression of antiapoptotic and antioxidant genes such as STAT3, Bcl-2, and B-cell lymphoma extra-large (Bcl-xL), as well as genes coding for AMPA and kainite receptor subunits. Moreover, quercetin decreased the levels of pro-inflammatory cytokines, such as IL-1β (Turovskaya et al., 2019). In conclusion, although the results from the application of quercetin in *in vitro* and *in vivo* neonatal HI models are quite promising, further studies in large animal models, as well as clinical trials are necessary for it to be considered as potential treatment for HIE.

### Pentoxifylline

Pentoxifylline (PTX), a methylxanthine derivative, is a non-selective phosphodiesterase inhibitor commonly used for the treatment of symptomatic vascular insufficiency because of its haemorrheological activity. In recent years, *in vivo* and *in vitro* studies have discovered that PTX also prevents or attenuates the release of TNF-α and other pro-inflammatory cytokines, underlying its potential therapeutic effects in HI.

Compared to administration of high PTX doses (100 mg/kg), intraperitoneal administration of low doses of PTX (60 mg/kg) provides significant protection against hippocampal atrophy and improves spatial learning and memory impairments in a rat HI model (Halis et al., 2019), thus suggesting hormetic effects. Such neuroprotection is believed to rely on PTX ability to reduce caspase 3 activity, as well as IL-1β and TNF-α-gene expression after a HI insult in P7 Wistar rats (Kalay et al., 2013). Moreover, pre-treatment with PTX markedly attenuated subsequent cerebral infarction and ischaemic forebrain injury after HI in P7 rats (Eun et al., 2000). Thus, there is potential for the use of PTX as treatment for neonatal HIE, however, further experiments are required to determine the precise dosage in large animal models and then in clinical trials.

### Oxymatrine

Oxymatrine (OMT) is a quinolizidine alkaloid extracted from the traditional Chinese herb *Sophora flavescens*. It has a tetracyclic quinolizine structure (Cells et al., 2013) and possess extensive pharmacological activities, including anti-inflammatory (Wang and Jia, 2014), anti-viral, hepatoprotective (Wen et al., 2014), anti-tumour (Liu D.-D. et al., 2014; Ying et al., 2015), immune-modulating, anti-oxidant (Wen et al., 2014), and anti-apoptotic features (Jiang et al., 2005; Hong-Li et al., 2008; Guo et al., 2014; Wen et al., 2014).

Intraperitoneal post-HI treatment of neonatal rats with OMT has provided neuroprotection by reducing the infarct volume and percentage of cell death, ameliorating histopathology and morphology of injured hippocampal neurons, increasing antioxidant enzyme activity [SOD, glutathione peroxidase (GSH-Px), and CAT], reducing lipid peroxide, as well as decreasing caspase-3 expression and increasing Bcl-2/Bax ratio (Zhao et al., 2015). Furthermore, OMT protects the rat brain from HI injury by reducing cell death possibly through down activation of NR2B and PI3K/Akt/GSK3β pathway (Liu et al., 2019). Due to the effective, non-toxic, and neuroprotective properties, OMT is considered to be a prospective preventive and restorative therapy for neonatal asphyxia in the clinical practice.

### Resveratrol

Resveratrol (RESV; trihydroxystilbene) is a natural non-flavonoid polyphenolic compound belonging to the phytoalexin superfamily, present in red wine/red grapes, soybeans, and pomegranates (Liu et al., 2007). It has two aromatic rings with three free hydroxyl groups which contribute to its free radical scavenging and antioxidant properties (Yousuf et al., 2009). RESV also exerts anti-inflammatory and anti-apoptotic effects and has been used to treat various illnesses including diabetes, cardiovascular and neurological diseases, and cancer (Karalis et al., 2011; Feng et al., 2016; Sadi and Konat, 2016).

Resveratrol positively modulates heme oxygenase 1 (HO-1) and nuclear factor erythroid 2 related factor 2 (Nrf2) protein expression, decreases infarct volume and cerebral oedema, elevates the levels of GPx and CAT, suppresses inflammatory markers, such as IL-1β, IL-6, TNF-α, and NF-κB, and improves neuronal survival after HI insult in the neonatal rat (Gao et al., 2018). Similar results were confirmed by Pan et al. (2016), where RESV ameliorated HI induced brain injury in parallel with reduction of Bax anti-apoptotic levels. Arteaga et al. (2015) showed that pre-treatment with RESV in a rat HI model reduced astroglial response, production of ROS and significantly decreased anxiety and neophobia (Arteaga et al., 2015). Pre- and post-HI treatment with RESV provides neuroprotection thus suggesting potential for its application as a therapy for HI.

### Pterostilbene

Pterostilbene (PTE) (3,5-dimethoxy-4-hydroxystilbene) is a natural compound found primarily in *Pterocarpus marsupium* heart wood and blue-berries (Adrian et al., 2000). PTE is a member of the phytoalexins family, which is produced in plants to defend against pathogens such as bacteria or fungi. Accumulative data suggests that PTE possesses various biological and pharmacological properties, including anti−oxidative, anti−inflammatory, anticancer and analgesic activities, and exerts neuroprotective effects under pathological conditions, such as ageing and Alzheimer’s disease (McCormack and McFadden, 2013).

Pterostilbene pre-treatment increases P7 rat survival, decreases brain infarct volume and brain oedema, attenuates the mRNA expression of TNF-α, IL-1β, IL-6, and p65 NF-κB, reduces programmed cell death and prevents oxidative stress by increasing SOD activity in HI-injured neonatal brain. Furthermore, intraperitoneal PTE injection improves motor coordination and deficit, and working memory impairment in a Sprague–Dawley rat HI model (Li D. et al., 2016). Thus PTE treatment could be potentially used for therapy in neonatal HIE.

### Erythropoietin

Erythropoietin (EPO), a 34 kDa glycoprotein cytokine, originally identified because of its role in promoting bone marrow erythropoiesis, has prompted a growing interest as neuroprotection agent in a series of neurological diseases. Its application in neonatal HI has improved the prognosis and is widely evaluated in experimental models and clinical trials (Villa et al., 2003; Xiong et al., 2011). To date, the possible mechanisms for EPO neuroprotection are associated with anti-apoptotic and anti-inflammatory properties, neurovascular remodelling, and promotion of neural stem cell proliferation (Xiong et al., 2011). HI in the brain leads to an increased EPO and EPO-R expression in neurons, astrocytes, and microglia, mediated by hypoxia-inducible factor-1 (Bernaudin et al., 1999, 2002; Mu et al., 2005). This upregulation represents an endogenous neuroprotective mechanism in the brain. Therefore, newborns with HIE show significantly elevated EPO levels in CSF, even in the absence of exogenous EPO treatment (Juul et al., 1999). Preclinical studies have shown that intraperitoneal EPO injection in P10 rat pups increased synaptic proteins Synapsin 1 and PSD95, thus improving synaptogenesis and spatial memory performance, and decreased neurite repair after HI insult (Xiong et al., 2019). EPO therapy can also protect P7 neonatal rat pups against HI brain injury by inhibiting Fas or FasL induced apoptosis (Huang et al., 2019) and by down-regulating metalloprotein kinase 2 (MMP-2), which in the adult brain is dramatically increased after cerebral HI (Zhang L. et al., 2017).

Phase II clinical trials of EPO administered without TH in the first week of life of neonates with HIE were safe and showed improvement in neurologic outcome (Zhu et al., 2009; Elmahdy et al., 2010). However, the studies were limited due to small sample size. In a larger randomised placebo-controlled phase III clinical trial, EPO administration decreased the risk of death and disability at a mean age of 19 months compared with placebo treated groups (Malla et al., 2017). A phase II clinical trial recruiting term neonates showed that high doses of adjunctive EPO treatment and TH may reduce MRI-assessed brain injury and improve motor function at 1 year post-HI (Wu et al., 2016). However, in severe HI cases such as in SOD-1 transgenic mice, EPO is not neuroprotective and worsens the injury as shown by Sheldon et al. (2017) possibly, because of interference with endogenous repair responses. Their findings suggest that when applied immediately after the insult, EPO treatment is not beneficial in cases of severe HI and extreme oxidative stress.

Overall, EPO is a very promising neuroprotective agent for HIE in term and preterm neonates The different proposed mechanisms underlying its neuroprotective effects are likely to be responsible for its early success in clinical trials. If the ongoing phase III trials demonstrate long-term neurodevelopmental benefit, EPO could be the first neuroprotective agent for preterm HIE outside of standard supportive care.

### Allopurinol

Allopurinol is a xanthine oxidase inhibitor, which inhibits the conversion of hypoxanthine into xanthine and uric acid in one of the main pro-oxidant pathways after HI, thereby limiting the toxic overproduction of ROS. Allopurinol’s anti-oxidant properties are based on the chelation of unbound iron and direct scavenging of free hydroxyl radicals. It prevents adenosine degradation and oxygen radical formation and preserves NMDA receptor integrity, so as a consequence it may reduce brain injury in HIE through several mechanisms of action (Pan et al., 2016; Gao et al., 2018).

In preclinical studies, subcutaneous allopurinol administration 15 min after HI in the P7 rat decreases brain oedema and selective neuronal necrosis (Gao et al., 2018). In combination with TH, allopurinol confers great functional, histological, and molecular neuroprotective effects (Rodríguez-Fanjul et al., 2017). Specifically, allopurinol treatment enhances neuropathological brain score, decreases cleaved caspase-3, and improves functional outcome after HI.

Phase I–III clinical trials suggest that postnatal allopurinol administration may provide neuroprotection to neonates with moderate HI brain damage (Gunes et al., 2007; Kaandorp et al., 2012). Antenatal administration of allopurinol to pregnant women may also attenuate hypoxic brain damage in female neonates with therapeutic levels detected in arterial cord blood, indicating successful placental crossing (Kaandorp et al., 2015). However, more trials and larger groups are needed to demonstrate the efficacy of allopurinol in preventing brain damage and improving outcome after neonatal HI insult.

### Indomethacin

Several studies have suggested that indomethacin, a non-selective inhibitor of prostaglandin synthesis, has a protective effect against anoxia and hypercapnia (Leffler et al., 1993; Ogasawara et al., 1999). Therefore, a potential therapeutic role of indomethacin in HI has been investigated. Indomethacin treatment in a rat HI model attenuated caspase activity and reversed glutathione depletion, thus providing neuroprotection. However, indomethacin also increased lipid peroxidation, which suggests that further investigation of its application in neonatal HI is needed (Taskin et al., 2009). To date, most of the pre-clinical evidence does not support the routine use of indomethacin in improving long-term neurodevelopmental outcome in preterm neonates.

### Topiramate

Topiramate is an AMPA/kainate receptor antagonist with multiple mechanisms of action, widely used as an anticonvulsant agent in adults and children (Shank et al., 2000; Guerrini and Parmeggiani, 2006).

In HI topiramate targets excitotoxicity during the secondary energy failure. Preclinical studies have shown that intraperitoneal topiramate injection in P7 rodent pups provides short-term neuroprotection by affecting GABA levels and improving learning ability after HI. However, in the long-term or when excessively used, topiramate may cause new CNS damage and reduce cognitive ability (Jiang et al., 2014). Interestingly, the combination of TH or memantine, a safe non-competitive low affinity NMDA receptor antagonist used in moderate to severe Alzheimer’s disease, with topiramate significantly reduced infarct volume in rodent and piglet HI models (Liu et al., 2004; Noh et al., 2006; Landucci et al., 2018). Phase I and II clinical trials in term neonates with HIE established the efficacy and safety of topiramate administration with and without concurrent TH (Filippi et al., 2010), suggesting therapeutic potential of that agent in neonatal HIE.

### Curcumin

Curcumin, a natural compound also known as diferuloylmethane (C_21_H_20_O_6_), is a major active component of the food flavour turmeric, isolated from the powdered dry rhizome of *Curcuma longa*. It is most frequently consumed in South Asian diets (Shishodia et al., 2005; Pescosolido et al., 2013). Except for turmeric usage as a dietary pigment, modern pharmacological studies show that curcumin provides therapeutic effects in several pathological conditions, such as cancer (Naksuriya et al., 2014; Ahmad et al., 2016), inflammation (Kim et al., 2003; Sandur et al., 2007), infections, cardiovascular diseases (Nishiyama et al., 2005; Liu and Hong, 2006), fibrosis, and neurological disorders (Spagnuolo et al., 2016), due to its anti-inflammatory, anti-oxidant, anti-apoptotic, anti-microbial, and ROS scavenging properties (Daugherty et al., 2018). As a result of its small molecular weight (368.385 g/mol) and dimensions, curcumin crosses the BBB (Priyadarsini, 2014) and was proposed as a possible treatment in different neurodegenerative disorders, such as Alzheimer’s (Reddy et al., 2016), Parkinson’s diseases, and multiple sclerosis (Wang et al., 2017).

Curcumin acts on many important pathways involved in the pathogenesis of HI injury (Panda et al., 2017). Specifically, it increases the levels of antioxidants such as SOD, GSH, and catalases, which are all implicated in free radical neutralisation (Alizadeh and Kheirouri, 2019). Also, curcumin inhibits the expression of pro-inflammatory cytokines (IL-1, IL-6, and TNF-α), thus mediating inflammation and inhibiting STAT3 phosphorylation (Maheshwari et al., 2006; Alexandrow et al., 2012). Recently, our group demonstrated that curcumin provides dose-dependent neuroprotection through immediate and delayed application following neonatal HI (Rocha-Ferreira et al., 2019). Two hundred micrograms per gram BW of curcumin reduced tissue loss, microglial and astroglial activation, and cell death after HI injury in a P7 mouse model. Prohibitin (PHB) is a protein considered essential in regulating mitochondrial structure and acting as a chaperone for the respiratory chain proteins. Curcumin administration post-HI increased PHB protein levels and provided neuroprotection through prevention of mitochondrial dysfunction during secondary energy failure (Rocha-Ferreira et al., 2019). Additionally, in a study conducted by Cui et al. (2017), curcumin was administrated to P7 rats at a dose of 150 mg/kg per day for 3 days, 24 h after induced HI-injury and resulted in prevention of myelin loss (Cui et al., 2017). Nrf2 provides neuroprotection (Zhang et al., 2015) and is elevated in curcumin treated mice. Curcumin treatment also significantly attenuates iNOS and caspase-3 expression when compared to untreated HI controls. Reduction of these pro-inflammatory and pro-apoptotic markers suggests that curcumin supresses inflammation and cell death in order to confer neuroprotection following neonatal HI. Due to its anti-inflammatory, anti-oxidant, and free scavenger properties, curcumin is considered to be a potential treatment for neonatal HI, but further preclinical studies are required to provide evidence for its efficacy.

### Melatonin

Melatonin is an endogenous indolamine hormone with anti-oxidant and anti-inflammatory properties, known for regulating the circadian rhythm (Claustrat and Leston, 2015). Preclinical models of HI demonstrate that melatonin is neuroprotective alone and as an adjuvant therapy with TH (Robertson et al., 2013, 2019; Carloni et al., 2014). Specifically, in conjunction with TH, melatonin significantly reduced cell death in a piglet HI model (Robertson et al., 2019, 2020), and decreased tissue loss and improved learning abilities in a rat HI model (Carloni et al., 2014). Combined with topiramate, melatonin significantly reduced infarction volume and number of TUNEL positive cells in a P7 rat HI model, suggesting that these agents may be beneficial for the treatment of infants with HIE (Ozyener et al., 2012). In a P7 HI rat model, three injections of 10 mg/kg melatonin within the first 25 h after injury provided only a transient and subtle reduction of infarct volume and behavioural impairment, but may not have been sufficient to mitigate long-term brain injury post-HI (Berger et al., 2016). The same group demonstrated that after HI injury in P7 rat pups melatonin was unable to protect neuronal mitochondria as indicated by GABA-A and lactate levels (Berger et al., 2019). Given its safety profile in animal models and the ease of crossing both the placenta and BBB, melatonin is a very attractive therapeutic candidate for HI. In a small prospective randomised trial, neonates with moderate to severe HIE were treated with melatonin. At 2 weeks of age neonates who received adjuvant melatonin showed fewer electrographic seizures detected by EEG and less white matter injury on brain MRI scans, compared to the neonates who received TH alone. At 6 months of age, the melatonin treated group had higher survival without neurodevelopmental abnormalities compared to the controls. An open-label dose escalation phase 1 clinical trial examining combined melatonin and TH treatment of term HIE is actively recruiting (NCT02621944). Although melatonin is a promising drug with a favourable safety profile, larger, randomised trials with neurodevelopmental outcome measured at a minimum of 18–24 months of age are required to establish a definitive therapeutic role for neonatal HIE.

### Hydrogen

Hydrogen (H_2_) therapy has been investigated as a potential therapeutic agent against HI injury due to its potency as anti-oxidant, anti-inflammatory, and anti-apoptotic agent (Htun et al., 2019).

Cai et al. (2008) demonstrated that H_2_ post-treatment of P7 HI rats reduced tissue loss, cell death, and caspase-3 and caspase-12 activity. The same study revealed that H_2_ treatment significantly reduced infarct volume and morphological neuronal damage associated with condensed cytoplasm and irregular cell shape, as well as AIF-1 expression as a marker of microglial inflammation. Furthermore, H_2_ treatment improves behaviour and cognitive function assessed through Morris water maze test for spatial learning and locomotor activity. Additionally, in a P7 rat HI model, H_2_ significantly attenuates neuronal injury and improves early neurological outcomes by reducing Bax and caspase-3 expression (Wang et al., 2020). In a piglet model of HI, H_2_ combined with TH, improved walking ability and decreased TUNEL positive cell death in dorsal cortex (Htun et al., 2019).

In a clinical study conducted by Yang et al. (2016), H_2_ reduced serum levels of the pro-inflammatory cytokines IL-6 and TNF-α, and neuron specific enolase (NSE) which can be used as a marker for nerve cell damage.

However, a study from Matchett et al. (2009) demonstrated that in moderate and severe HI rat models, hydrogen gas therapy did not decrease infarct volume or the concentration of malondialdehyde (MDA), an end-product of lipid peroxidation. In conclusion, there is no effect of H_2_ treatment in moderate and severe HI models, so further studies are necessary to establish whether H_2_ provides necessary neuroprotection for HIE.

### Magnesium

Magnesium (MgSO_4_) is an ionised mineral essential for hundreds of enzymatic processes, including hormone receptor binding, energy metabolism, and muscle contractility (Solevåg et al., 2019). It is also an NMDA receptor antagonist which prevents excitotoxic calcium-induced injury through the voltage-dependent inhibition of the NMDA receptor, thus reducing calcium entry into the cell (Ovbiagele et al., 2003). As a result, several injurious pathways, implicated also in HI, including catabolic enzyme induction and increased ROS production are prevented (Lingam et al., 2019). Magnesium also inhibits NF-κB thus providing anti-inflammatory effects (Lingam et al., 2019).

Pre-treatment with MgSO_4_ 6 days to 12 h prior to HI in P7 rats reduces the neonatal brain injury and attenuates ROS production and post-HI accumulation of chemokines and pro-inflammatory cytokines (IL-1α, IL-1β) (Koning et al., 2019). Additionally, MgSO_4_ pre-HI treatment also downregulated metabolic pathways including mitochondrial network genes, especially those corresponding to proteins in the electron transport chain (complex I and II) (Koning et al., 2019).

Post-HI MgSO_4_ treatment in P7 rats alone or in combination with melatonin, significantly reduced hippocampal infarct volume and cell death, indicating that these agents may confer a possible benefit in the treatment of infants with HI (Cetinkaya et al., 2011). These results were confirmed in a piglet HI model, where MgSO_4_ combined with TH reduced cell death and increased oligodendrocyte survival in hippocampus and thalamus (Lingam et al., 2019). Spandou et al. (2007) demonstrated that magnesium treatment in a P7 rat model of moderate HI (1 h hypoxia) reduced brain damage and increased ATP and glutamine levels, but did not prove neuroprotective when the animals were subjected to severe, 2 h, hypoxia. The lack of neuroprotection following MgSO_4_ application has been also demonstrated in a P7 HI rat model, where post-HI MgSO_4_ treatment failed to improve striatal neuronal survival (Galvin and Oorschot, 1998). This lack of neuroprotection was also confirmed in a piglet HI model, where MgSO_4_ treatment resulted in no difference in the severity of damage in hippocampus, cerebellum, cerebral cortex, caudate nucleus, thalamus, striatum, and white matter tracts (Greenwood et al., 2000). Magnesium has been also investigated in clinical trials and especially as an antenatal strategy for preterm HI. The outcome of magnesium infusions demonstrated a lower incidence of cerebral palsy in infants (Doyle et al., 2009). Moreover, combined therapy of MgSO_4_, erythropoietin, and TH proved to be safe in an open-label pilot study investigating the feasibility of combining therapeutics in HI patients (Nonomura et al., 2019).

Overall, magnesium is a promising antenatal therapeutic strategy for preterm HI and given its low cost and availability is considered standard care for mothers at risk for preterm delivery (Doyle et al., 2009). However, larger clinical trials are needed to provide evidence for its efficacy in term delivery.

### Coumestrol

Coumestrol, a potent isoflavonoid with oestrogen-like structure and actions, is present in soy beans, clover, peas, and alfalfa, and is well-known for its multiple biological features, including antioxidant (Koirala et al., 2018) and anti-inflammatory (You et al., 2017) properties. In P7 rats pre-HI treatment with coumestrol prevented mitochondrial failure, as shown by the decrease of MitoTracker Red (MTR) and MitoTracker Green (MTG) ratio. These markers are widely used to reveal the mitochondrial membrane potential and mitochondrial mass, respectively. Furthermore, both pre- and post-HI application of coumestrol counteracted spatial orientation and working memory impairments assessed through Morris water maze test (Anastacio et al., 2019). Moreover, coumestrol treatment reduces tissue loss and blocks long-term reactive astrogliosis (Anastacio et al., 2019) suggesting potential for treatment of HIE.

### Xenon

Xenon is a noble, colourless, odourless gas that is four times heavier than oxygen. It has been used as a safe and efficient anaesthetic since 1951 (Amer and Oorschot, 2018). Trials in human infants show that Xenon is hemodynamically safe (Dworschak, 2008; Faulkner et al., 2011) and that it crosses the BBB (Dworschak, 2008). Xenon reduces hypoxic brain injury following HIE and stroke in neonatal rat and piglet models (Chakkarapani et al., 2010; Faulkner et al., 2011; Sheng et al., 2012).

In preclinical studies, Xenon up-regulates anti-apoptotic proteins (Bcl-2) and the Bcl-xL mitochondrial membrane molecule, modulates pro-inflammatory cytokine levels (TNF-α) thus decreasing inflammation, and increases growth-factors (VEGF) leading to reduced cell death and enhanced repair (Amer and Oorschot, 2018). Xenon combined with TH in a P7 rat HI model, improves behavioural outcome assessed through staircase test (Osredkar et al., 2014).

Low Xenon concentration combined with mild TH, both not showing neuroprotection alone, had a synergistic neuroprotective effect in a moderate P7 HI rat model when treatment with both agents was initiated at 4 h following the insult (Ma et al., 2005). However, these results were not confirmed (Sabir et al., 2014). Sabir et al. (2016) observed no change in brain area loss and neuronal cell count in any of the experimental groups, thus demonstrating lack of neuroprotection when combining Xenon and TH in a severe HI P7 rat model.

In line with the promising preclinical studies, a small, dose escalation feasibility study was conducted in neonates with moderate or severe HIE receiving TH. Inhalation of 50% Xenon/50% oxygen reduced electrographic seizures, increased sedation, and diminished EEG background without blood pressure reduction in all participating neonates. At 18- to 20-month follow-up, the developmental outcomes were no worse than TH treatment alone (Dingley et al., 2014). Subsequently, a larger feasibility and safety trial was completed where neonates with moderate or severe HI were treated with TH alone, or with TH and inhaled 30% Xenon/70% oxygen for 24 h. The combination of TH and Xenon did not provide additional protection in respect to mortality or early brain injury assessed through MRI, when compared to TH alone (Azzopardi et al., 2016). The high cost and specialised delivery systems make Xenon less likely to be widely implemented. The extent of neuroprotection from inhaled Xenon for neonates with HIE, as well as the optimal timing, dosing, and feasibility of broad administration, remain to be determined.

### Umbilical Cord Blood Cells, Stem Cells, and Extracellular Vesicles

Umbilical cord blood cells (UCBCs) possess immunomodulatory properties leading to suppression of inflammation (Pimentel-Coelho et al., 2012) and their transplantation has proven neuroprotective in a range of preclinical CNS injury models (Kang et al., 2015; Li J. et al., 2016). As UCBCs are readily available at the time of birth, they pose an especially attractive therapeutic potential for HI. Moreover, elevated lactate levels in umbilical cord blood (UCB) samples of infants with birth asphyxia is a potential marker for early prediction of HI injury (Anh et al., 2019). Therefore, in suspected cases of HI injury, combining testing and treatment with UCB extracted from the placenta could be a promising approach.

Umbilical cord blood mononuclear cell fractions contain an array of cell types that individually or together could be responsible for the therapeutic effects observed in preclinical studies. These are haematopoietic stem/progenitor cells (HPCs), mesenchymal stromal cells (MSCs), endothelial progenitor cells (EPCs), regulatory T-cells (Tregs), monocytes, and lymphocytes (Pimentel-Coelho et al., 2012).

Administration of human UBC mononuclear cells, EPCs, and Tregs in a P7 rat HI model, reduced Iba-1 expression as a marker of microglial activation, and provided neuroprotection. Furthermore, only treatment with EPCs significantly reduced cell death. Following HI injury, as a consequence of the inflammatory response, the levels of infiltrating CD4+ T-cells in the brain are elevated. Treatment with human UCB mononuclear cells, Tregs, and monocytes significantly reduced the levels of CD4+ T cells (McDonald et al., 2018). In a rat P8 HI model, treatment with human UCBC improved long-term behavioural outcomes assessed through open field test, cylinder test, and negative geotaxis (Penny et al., 2019).

In a recent clinical study by Tsuji et al. (2020), six newborns with severe birth asphyxia were intravenously dosed with autologous UCBCs alongside TH (Tsuji et al., 2020). After 18 months, four of the treated infants displayed normal neurodevelopment and two presented with cerebral palsy, however, no adverse effects from the cell transplantation therapy were observed, deeming the treatment protocol alongside TH to be both safe and feasible.

Mesenchymal stromal cells participate in the maintenance of homoeostasis and restoration of tissue after injury through secretion of soluble factors and extracellular vesicles (EVs). EVs (exosomes and microvesicles) are 30–1000 nm lipid bilayer-enclosed structures released from parental cells and participating in cell-to-cell signalling processes. EVs transport various biologically active molecules such as proteins, mRNAs, miRNAs, lncRNAs, DNA, and lipids to target cells (Inal et al., 2012; Yeo et al., 2013; György et al., 2015; Bruno et al., 2017; Tricarico et al., 2017; Van Niel et al., 2018). Anti-inflammatory factors are a key group of molecules released by MSCs, and are important in mediating repair (Drago et al., 2013; English, 2013; Madrigal et al., 2014). HI studies using MSCs as putative treatment demonstrated neuroprotective potential for those cells (van Velthoven et al., 2010; Kim et al., 2012; Donega et al., 2014; Ahn et al., 2016; Corcelli et al., 2018). Moreover, the therapeutic time window was extended when MSC application was combined with TH (Ahn et al., 2018). Post-HI treatment with MSC-derived EVs in P7 mice significantly reduces microglial activation, cell death, and tissue loss and improves behavioural outcomes (Sisa et al., 2019b). Post-HI treatment with MSC-derived EVs was also neuroprotective in preterm ewes, since it prevented loss of cortical function assessed through EEG, and reduced white matter injury (Ophelders et al., 2016).

### Diabetes Drugs

Over the past decade, pre-clinical, and clinical studies have provided evidence that drugs treating diabetes are neuroprotective in different neurological conditions, such as Alzheimer’s disease, stroke, and epilepsy (Athauda et al., 2017; Rotermund et al., 2018; Mousa and Ayoub, 2019). The effectiveness of some diabetes drugs, such as metformin, sulphonylurea, and incretin/glucagon-like peptide-1-receptor (GLP1-R) agonists, has been investigated in neonatal HI.

Metformin, is a biguanide widely used for the therapy of type 2 diabetes mellitus and metabolic syndrome. Metformin exhibits a diverse range of pharmacological characteristics, such as anti-oxidant, anti-inflammatory, anti-apoptotic, anti-tumour properties (Ashabi et al., 2015; Eikawa et al., 2015). Recently, metformin was reported to exert neuroprotective effects in a variety of animal models of CNS diseases including HI, *via* regulation of the inflammatory response, neuronal apoptosis, and oxidative stress (Liu Y. et al., 2014; Ge et al., 2017; Zhang D. et al., 2017). Metformin treatment in a P7 HI mouse model, significantly attenuated brain damage, by reducing pro-inflammatory factors (TNF-α, IL-1β, and IL-18), decreasing micro- and astro-glial activation, attenuating TUNEL positive cell-death, and by ameliorating infarct volume and brain oedema (Fang et al., 2017).

Sulphonylurea agents are hypoglycaemic drugs, with their receptor, sulphonylurea receptor 1 (SUR1) being involved in brain injury in rodent models of stroke (Hussien et al., 2018). KATP is a microglial channel, which is overexpressed in rodent models of stroke (Hussien et al., 2018). SUR1 is a regulatory subunit of KATP. Drugs blocking SUR1, and especially glibencalmide, exert neuroprotective effect. This could be attributed to inhibition of microglia activation, which, if initiated, will cause release of pro-inflammatory cytokines and will start downstream signalling pathways, resulting in neuronal cell death (Ortega et al., 2013). In a rat model of HI, glibencalmide improved motor performance assessed through postural reflex test (Zhou et al., 2009).

Glucagon-like peptide-1-receptor agonists, such as liraglutide and exendin-4, are used in combination with diet and exercise for treatment of type 2 diabetes. They also provide neuroprotection in rodent models of epilepsy and stroke (Wen et al., 2019). Treatment with liraglutide after HI brain injury in P7 rats, attenuated infarct volume and cell oedema, decreased TNF-α levels, reduced tissue and neuronal loss, enhanced axonal repair and accelerated re-myelination (Hussien et al., 2018; Zeng et al., 2020). Liraglutide provides neuroprotection *via* PI3K/Akt pathway (Zeng et al., 2020). Application of exendin-4 alone or in conjunction with TH in a neonatal mouse HI model also provided neuroprotection (Rocha-Ferreira et al., 2018). In conclusion as quite a few studies support the anti-inflammatory and neuroprotective effects of specific diabetic drugs in neonatal HI either independently or in combination with TH, their further investigation as treatment for the condition is justified.

### Osteopontin

Osteopontin (OPN) is a glycoprotein hormone synthesised by various tissues and present in all body fluids (Denhardt et al., 2001). OPN expression has both pro- and anti-inflammatory properties, and is mediated through regulation of various cytokines (IL-10, IL-12, IL-3, and Interferon-γ), NF-κB, macrophages, and T cells (Icer and Gezmen-Karadag, 2018).

Evidence of the importance of OPN in neuronal protection post-HI injury was demonstrated in an OPN knockout mouse model, where P9 mice subjected to HI insult developed greater loss of grey and white matter, and more pronounced sensorimotor deficits (Van Velthoven et al., 2011). OPN-deficient mice also displayed less cerebral cell proliferation, survival, and oligodendrogenesis, thus supporting a pivotal role for OPN in brain injury, particularly in white matter recovery post-HI.

Alternatively, exogenous OPN administration through intracerebroventricular injection following HI in P7 rats decreased infarct volume, reduced cell death, and improved behavioural performance assessed at 7 weeks post-HI using Morris water maze (Chen et al., 2011).

However, a study from Bonestroo et al. (2015b) demonstrated that intravenous administration of TAT-OPN peptide in a P9 HI mouse model did not improve brain injury or sensorimotor behavioural deficits, and caused no functional improvement (cylinder rearing test and adhesive removal task) or decrease of cerebral damage (Bonestroo et al., 2015b). Thus, as the supporting evidence for the neuroprotective effects of OPN in neonatal HI is not very strong and the data are controversial, further pre-clinical investigations are required.

### C-Jun N-Terminal Kinases

C-Jun N-Terminal Kinases (JNKs) are protein kinases participating in stress signalling pathways. For example, neuronal apoptosis is mediated *via* downstream phosphorylation of c-Jun by JNK leading to apoptotic cell death in HI (Mielke and Herdegen, 2000). JNKs are activated in response to inflammation and excitotoxicity (Benakis et al., 2010).

Pirianov et al. (2007) demonstrated that deletion of JNK3 in a P9 HI mouse model substantially reduced neuronal tissue loss, attenuated c-Jun phosphorylation and the expression of adenovirus transcription factor-2 (ATF-2), which is involved in apoptosis, implicating a critical role for JNK3 in neuronal cell loss following HI insult. Similarly, Nijboer et al. (2013) showed reduced brain damage in P7 HI rats treated with the JNK inhibitor TAT-JBD. Likewise, D-JNKi, an inhibitor of mitochondrial JNK phosphorylation, reduced neuronal damage and enhanced cognitive and sensorimotor function in P7 HI rats (Nijboer et al., 2013).

More recently, the role of JNK in cell death and HI was further emphasised in a study showing that inhibition of apoptosis signal-regulating kinase 1 (ASK1), involved in JNK phosphorylation and activation, confers neuroprotection (Hao et al., 2016). Intracerebroventricular injection of NQDI-1, a specific inhibitor of ASK1, was applied in P7 female rats post-HI insult. This resulted in lower expression of phosphorylated ASK1, JNK, c-Jun, p53, and caspase 3, and reduced brain infarct volume and cell death (Hao et al., 2016). Collectively, these studies support the importance of JNK signalling in HI injury and cell death, and highlight it as a novel therapeutic target.

### Edaravone

Edaravone (3-methyl-1-phenyl-2-pyrazolin-5-one) is a novel synthetic free radical scavenger and has been clinically used to treat patients with acute brain infarction since 2001 (Higashi et al., 2006). Edaravone, as a result of its amphiphilicity, was designed to scavenge both lipid and water soluble peroxyl free radicals, along with other ROS species (Watanabe et al., 2018), therefore suggesting a potential protective role in neonatal HI injury.

Pre-HI intraperitoneal treatment with edaravone in P7 rat pups reduced caspase-3 levels, and therefore decreased cell death (Yasuoka et al., 2004). These results were confirmed by Takizawa et al. (2009), in parallel with reduced DNA peroxidation/oxidative stress. Post-HI edaravone treatment in P7 mice reduced lipid peroxidation by-products (Noor et al., 2005a). Furthermore, edaravone treatment significantly decreased nitric oxide metabolites in the CSF collected before the mice were culled. As lipid peroxidation and oxidative stress are increased in the pathophysiology of neonatal HI injury, and edaravone counteracts them, these results support a protective role for that compound in neonatal HI.

A study by Li et al. (2018), demonstrated that edaravone treatment in a P7 rat HI model, significantly downregulated the expression of FADD, caspase 8, and DR5 apoptotic markers after HI. In the same study edaravone treatment also reduced caspase-3 expression, suggesting suppression of apoptosis and therefore improving neurofunctional performance in Morris water maze test (Li et al., 2018).

A study by Noor et al. (2005b) in a P7 Wistar rat HI model, showed that edaravone was neuroprotective only to the acute phase (two consecutive days of administration) after HI by improving learning and memory capability as well as morphological brain recovery, but was not effective after 5 or 10 consecutive days of administration. A recent study in a piglet HI model (24 h-age piglets but they don’t specify the day of surgeries) demonstrated that intravenous administration of edaravone combined with TH did not improve neurological outcomes in grey or white matter, nor attenuated hippocampal brain damage (Yamato et al., 2020). Other methods of drug administration are necessary to address the efficacy of combined endaverone and TH treatment for neonatal HI brain injury.

### Granulocyte-Colony Stimulating Factor

Granulocyte-colony stimulating factor (G-CSF) is an endogenously produced haemopoietic growth factor, known for its immunomodulating properties, primarily acting in an anti-inflammatory way (Hartung, 1998). Preclinical studies looking at the use of G-CSF for therapeutic benefits in neonatal HI has shown some promise.

Yata et al. (2007) tested 1 h delayed G-CSF administration in a P7 HI mouse model, and observed reduced tissue loss, as well as decrease in TUNEL positive cell death and Bax and caspase-3 proteins, indicating that G-CSF attenuated apoptosis and neuronal loss.

Long-term neurological function including short-term memory, motor coordination, reflexes, and exploratory behaviour improved after G-CSF treatment in a P7 rat HI model (Fathali et al., 2010). G-CSF treatment in a model of perinatal hypoxia in P7 rats, also rescued long-term cognitive function, suggesting protection against degeneration in hippocampus, midbrain, and temporal cortex (Yang et al., 2013d).

Most recently, Dumbuya et al. (2020) demonstrated that G-CSF treatment in P7 HI rats reduced apoptosis and promoted the expression of IL-10. Simultaneously, G-CSF treatment also decreased infarct volume and tissue loss, and reduced expression of caspase-3, Bax, and Bcl-2. Moreover, the expression of the mTOR/p70S6K pathway was downregulated in the G-CSF treated group, in combination with reduction in the expression of TNF-α and IL-1β, and in TUNEL positive cells. Overall, G-CSF treatment demonstrated anti-apoptotic and anti-inflammatory properties after HI insult and improved behavioural outcomes making it a potential candidate for HI treatment. However, studies on larger animal models and clinical trials are needed to establish its efficacy.

### Anti-inflammatory Cytokines

Anti-inflammatory cytokines protect neurons against HI caused hyper-excitability and death *in vitro* and *in vivo* (Turovsky et al., 2013; Tukhovskaya et al., 2014). In HI neuronal cultures, IL-10 suppresses re-oxygenation triggered hyper-excitability through inhibition of Ca^2+^ release from the endoplasmic reticulum, delay of global Ca^2+^ increase and promotion of cell survival (Turovskaya et al., 2012; Turovsky et al., 2017). PI3-kinase inhibition abolishes the neuroprotective effects of IL-10 (Turovskaya et al., 2014). This suggests that the protection provided by IL-10 during ischaemia is mainly mediated by PI3-kinase-dependent cell survival signalling pathways (Sharma et al., 2011). *Sip1* is a transcription factor involved in neurogenesis regulation, and its mutation leads to suppressed expression of genes encoding the subunits of NMDA, AMPA, and kainate receptors; protein kinases PKA, JNK, CaMKII, as well as transcription factor Hif1α, thus causing postnatal microcephaly and epileptic seizures. In neuronal mouse cell cultures with the *Sip1* mutation IL-10 treatment restores neurotransmission by increasing the expression of the above mentioned genes, although not to the levels of wild-type controls (Turovskaya et al., 2020). Overall, IL-10 provides neuroprotection *in vitro*, however, further studies *in vivo* are needed to confirm its role in HI conditions.

A summary of the current neuroprotective agents for neonatal HI brain injury used in pre-clinical studies and in clinical trials is shown in Table 1.

**TABLE 1:** Summary of neuroprotective agents for neonatal brain injury in pre-clinical studies and in clinical trials.

Agent	Preclinical evidence	Clinical trials	Negative results
Cannabinoids	• Decreases inflammation, excitotoxicity, oxidative stress in the rat (Pazos et al., 2013). • In the mouse and piglet, reduced astroglia activation and tissue loss (Martínez-Orgado et al., 2007; Mohammed et al., 2016).	–	• In a piglet model.
Quercetin	• Decreases microglial, astroglial activation, apoptotic markers in the rat (Wu et al., 2019). • Increases oligodendrocytes proliferation. Improves spatial and memory learning and cognitive ability in the rat (Huang et al., 2012; Qu et al., 2014).	–	–
Pentoxifylline	• Decreases hippocampal atrophy, apoptotic markers, inflammation markers in the rat (Kalay et al., 2013). • Improves spatial learning and memory in the rat (Halis et al., 2019).	–	–
Oxymatrine	• Reduces infarct volume, apoptosis, and oxidative stress in the rat (Zhao et al., 2015; Liu et al., 2019). • Ameliorates morphology of injured hippocampal neurons in the rat (Zhao et al., 2015).	–	–
Resveratrol	• Decreases infarct volume, cerebral edoema, apoptosis, elevates anti-oxidative enzymes activity, reduces pro-inflammatory markers in the rat (Pan et al., 2016; Gao et al., 2018). • Reduces astrogliosis and improves behavioural outcomes (anxiety and neophobia) (Arteaga et al., 2015).	–	–
Pterostilbene	• Decreases infarct volume, apoptosis, and pro-inflammatory markers; improves motor coordination, working memory deficit in the rat (Li D. et al., 2016).	–	–
Erythropoietin	• Improves synaptogenesis, reduces apoptosis, improves spatial memory in the rat (Zhang L. et al., 2017; Huang et al., 2019; Xiong et al., 2019).	• Successful phase I, II, and III clinical trials completed as monotherapy application (Zhu et al., 2009; Elmahdy et al., 2010; Malla et al., 2017). • Active phase II clinical trial as augmentation with TH (Wu et al., 2016). • Active phaseIII clinical trial as augmentation with TH (Sheldon et al., 2017).	• In severe HI injury EPO worsens the outcome because it interferes with endogenous repair responses (Sheldon et al., 2017).
Allopurinol	• Decreases acute brain edoema and sub-acute brain atrophy in the rat (Palmer et al., 1993). • Decreases caspase-3 mediated apoptosis in the rat (Rodríguez-Fanjul et al., 2017).	• Successful postnatal clinical trials I–III as monotherapy (Gunes et al., 2007; Kaandorp et al., 2012). • Active postnatal phase III trial as augmentation with TH. • Successful phase III trial on antenatal administration (Kaandorp et al., 2015).	–
Indomethacin	• Reduced caspase mediated apoptosis, glutathione depletion, and lipid peroxidation in the rat (Taskin et al., 2009).	–	–
Topiramate	• The acute administration reduces histopathological brain injury and improves behavioural outcomes (Jiang et al., 2014; Landucci et al., 2018) in rodents. • Reduces infarct volume in augmentation with TH in the piglet (Noh et al., 2006).	• Successful safety phase I trial as monotherapy. • Successful phase I and II trials as augmentation with TH. • Active further augmentation phase I and II trials (Filippi et al., 2010).	–
Curcumin	• Decreased microglia, astroglia activation, cell death, and tissue loss if administered up to 2 h after HI insult in the mouse (Rocha-Ferreira et al., 2019). • Improved myelination and reduced iNOS levels in the mouse (Rocha-Ferreira et al., 2019). • Increased expression of nuclear factor erythroid-2-related factor 2 (Nrf2), attenuation of the increased expression of inducible NOS, and caspase-3 activity in the rat (Cui et al., 2017).	–	–
Melatonin	• If administered with TH, decreased tissue loss and improved learning in the Morris Water-Maze test in the rat (Carloni et al., 2014). • Reduction of cell death if administered in augmentation with TH in the piglet (Robertson et al., 2019, 2020). • If administrated with topiramate reduced infarct volume and cell death in the rat (Ozyener et al., 2012).	• Successful phase II augmentation trial with TH. • Active phase I augmentation trial with TH (NCT02621944).	• Only subtle neuroprotective effect but not long-term brain injury improvement in the rat (Berger et al., 2019). • No protection of neuronal mitochondria as shown by GABA-A and lactate levels (Berger et al., 2016).
Hydrogen	• Reduces cell death *via* reduction of caspase-3 and 12 activity, infarct volume, inflammation *via* AIF-1 expression reduction in the rat (Cai et al., 2008). • Improves spatial learning measured *via* Morris Water maze and locomotor activity in the rat (Wang et al., 2020) and piglet (Htun et al., 2019).	• Clinical study showed reduction of IL-6 and TNF-α cytokines (Domoki, 2020).	• Not associated with decreased infarct volume or decreased concentration of malondialdehyde (MDA), an end-product of lipid peroxidation in the rat (Matchett et al., 2009).
Magnesium	• If administered prior HI insult it reduces ROS production, IL-1α and IL-1β, and overall cell metabolism in the rat (Koning et al., 2019). • If administered in adjunction with melatonin, it reduces infarct volume of hippocampus and cell death in the rat (Cetinkaya et al., 2011). • If administered with TH reduces infarct volume of hippocampus, cell death and increases oligodendrocytes survival in hippocampus and thalamus in the piglet. • Inconsistent neuroprotection in rodent models.	• Clinical study showed lower incidence of cerebral palsy in infants (Doyle et al., 2009). • An open-label pilot study showed that combination of MgSO_4_, erythropoietin and TH was found to be safe (Nonomura et al., 2019).	• Not neuroprotective when administrated to rat after severe HI (Galinsky et al., 2014). • Post-injury treatment did not improve neural survival in striatum in rat (Galvin and Oorschot, 1998). • Post-HI treatment did not show any difference in the severity of damage on hippocampus, cerebellum, cerebral cortex, caudate nucleus, thalamus, and striatum and the white matter tracts in the piglet (Greenwood et al., 2000).
Coumestrol	• Pre-treatment prevents mitochondrial failure, improved spatial reference and working memory, reduced tissue loss and long-term astrogliosis in the rat (Anastacio et al., 2019).	–	–
Xenon	• Upregulaition of Bcl-2 and Bcl-xL improving apoptosis, reduction TNF-α and VEGF enhancing cell repair and reducing inflammation in the rat (Amer and Oorschot, 2018). • Improves motor function in the staircase test in the rat in augmentation with TH (Osredkar et al., 2014).	• Successful augmentation trial with TH in reducing apoptosis and cerebral abnormalities (Dingley et al., 2014). • Failed to show improvement compared to TH in moderate and severe cases (Azzopardi et al., 2013).	• Xenon combined with TH is not neuroprotective after severe HI in a P7 rat model since brain area loss and neuronal cell count were similar in all experimental groups (Sabir et al., 2016).
UCBs/MSCs	• Reduce iba-1, CD4+ T cells and improve locomotor activity measured with open field test, cylinder test, and negative geotaxis tests in the rat (Penny et al., 2019). • Reduced microglia, cell death, tissue loss in the mouse (Sisa et al., 2019b). • Prevention of cortical loss and function measured *via* EEG and reduced white matter injury in ewes (Ophelders et al., 2016).	• Small open label clinical study showed safety and feasibility as augmentation with TH (Tsuji et al., 2020).	–
Diabetes drugs	• Metformin reduced TNF-α, IL-1β, IL-18, microglia, astroglia activation, cell death, and tissue loss in the mouse (Fang et al., 2017). • Glibencalmide improves neuromotor activity in the rat (Zhou et al., 2009). • Liraglutide attenuated the infarct volume and cell oedema, decreased the inflammatory response at TNF-α levels, reduced tissue, neuronal loss, enhanced axonal repair, and accelerated remyelination (Zeng et al., 2020).	–	–
Osteopontin	• Increased cell proliferation, oligodendrogenesis; Decreases infarct volume, cell death; improves behavioural outcomes in the mouse (Van Velthoven et al., 2011), • Decrease infarct volume, reduced cell death and improve memory *via* MWM in the rat (Chen et al., 2011),	–	• TAT-OPN peptide did not exert neuroprotective effects on neonatal HI-induced brain injury or sensorimotor behavioural deficits in a mouse (Bonestroo et al., 2015b).
C-Jun N-terminal kinases	• Reduces neuronal loss, cell death, apoptosis in the mouse (Pirianov et al., 2007). • Enhances cognitive and sensorimotor function, reduces apoptosis, reduces brain infarct volume in the rat (Nijboer et al., 2013; Hao et al., 2016).	–	–
Edaravone	• Pre-treatment in the rat and the mouse down-regulates cell death, oxidative stress, apoptosis markers, lipid-peroxidation by-products (Yasuoka et al., 2004; Takizawa et al., 2009).	–	• Post-HI treatment is neuroprotective only to the acute phase after HI but not 5–10 days after insult in a rat (Takizawa et al., 2009). • Intravenous administration in combination with TH did not improve neurological outcomes in the newborn HI piglet as indicated by grey, white matter, and hippocampal brain damage (Yamato et al., 2020).
Granulocyte-colony stimulating factor	• Decreases cell death, tissue loss, apoptosis, inflammation in the mouse and rat (Yata et al., 2007; Dumbuya et al., 2020). • Improves long-term cognitive function and exploratory behaviour in the rat (Yang et al., 2013d).	–	–
Anti-inflammatory cytokines	• IL-10 increases cell survival and restores neurotransmission in neuronal cell cultures in ischaemic conditions (Tukhovskaya et al., 2014; Turovsky et al., 2017).		

## Experimental Treatments for Infection-Sensitised HI

### Histone Deacetylase Inhibitor Trichostatin A

Histone deacetylase (HDAC) works in synergy with acetyltransferase to regulate protein acetylation through post-translational modifications of histones or other proteins thereby modulating gene expression (Adcock, 2007). Histone deacetylase inhibitor (HDACi) treatment in adult rodent models of ischaemic/reperfusion stroke reduced the expression of pro-inflammatory molecules such as p53 and NF-κB (Hyeon et al., 2007; Shein et al., 2009). Moreover, *in vitro* exposure to HDACis reduces LPS-induced inflammation by repressing inflammatory cell recruitment and cytokine expression (Brogdon et al., 2007; Suh et al., 2010). Intraperitoneal administration of trichostatin A (TSA), a class I/II HDACi, in P7 HI mice pre-sensitised with LPS led to increased histone acetylation which persisted for 24 h after injury, reduced white and grey matter injury as well as improved long-term learning in female mice (Fleiss et al., 2012). TSA treatment did not provide neuroprotection in male mice, which can be attributed to the endogenously higher histone acetylation observed in male mice, thus suggesting less unacetylated lysine residues availability in males (Tsai et al., 2009). Whilst ample studies have shown that class I/II HDACis are neuroprotective in adult animal models through modulation of inflammation-associated molecules (Chen et al., 2007; Hyeon et al., 2007; Sinn et al., 2007; Wu et al., 2008), little is known of the mechanisms of action of HDACis in neonates. Even though Fleiss et al. (2012), observed neuroprotection in female mice, reduction of pro-inflammatory cytokine expression in the LPS/HI neonatal mouse was not registered, suggesting that the effect of HDACis does not mediate inflammation, but rather involves caspase-3 and Heat shock proteins (Liu et al., 1989; Yakovlev et al., 2010).

### Plasminogen Activator Inhibitor-1

Tissue-type plasminogen activator (tPA) is a serine protease circulating in the blood and brain parenchyma, widely known for its role in fibrinolysis (Gualandris et al., 1996). In the CNS, tPA is involved in various plasminogen-independent pathways where it potentiates ischaemia-induced excitotoxity by modulating NMDA receptor signalling (Nicole et al., 2001) and increasing production of nitric oxide (Parathath et al., 2006), as well as impairing BBB integrity (Su et al., 2008). tPA affects microglial activation (Rogove et al., 1999) through binding to the low-density lipoprotein receptor-related protein-1 (LRP-1) which leads to NF-κB activation – this is suppressed by preventing tPA-LRP-1 interactions after focal ischaemia in adult animals (Zhang et al., 2007, 2009). Earlier studies by Yang et al. (2009) in a rat model of HI injury, show that antagonising tPA activity with plasminogen activator inhibitor-1 (PAI-1) decreases HI-induced tPA activation and brain damage. Given the group’s previous work in pure HI and the involvement of tPA in microglial activation, Yang and colleagues investigated a stable-mutant form of PAI-1 called CPAI in LPS-sensitised P7 HI Wistar rats. Both intracerebroventricular (ICV) (Yang et al., 2013b) and intranasal CPAI delivery (Yang et al., 2013c), the latter considered a more clinically favourable administration route; showed similar efficiency in LPS-sensitised HI injury. ICV CPAI administration to LPS-sensitised HI rats reduced BBB damage, as well as decreased TNF-α and MCP-1 levels indicating a suppression in microglial activation. Moreover, CPAI treatment appeared to lower abnormal white matter development and motor impairments. Together, these results indicate a therapeutic role for PAI-1 in both HI alone and LPS-sensitised HI.

### Cell-Penetrating Anti-NF-κB Peptides

Acute activation of NF-κB plays a critical role in LPS-sensitised HI brain injury (Yang et al., 2013a) and its inhibition might provide a useful therapeutic intervention. Such inhibition can be achieved with a selective NF-κB inhibitor, anti-NF-κB peptides (Tat-NBD), comprised of the NF-κB essential modifier-binding domain peptide (NEMO) coupled with the HIV trans-activator of transcription peptide (HIV-TAT) (May et al., 2000; Pizzi et al., 2009). Yang et al. (2013a) used Tat-NBD to intranasally treat postnatal day 7 rats at 10 min after HI, and attenuated the brain damage with both 4 or 72 h LPS pre-HI exposure; the latter being more reflective of intrauterine infection. Tat-NBD treatment in both models reduced NF-κB and decreased microglial activation. Brain atrophy in HI animals pre-exposed to LPS for 4 h showed an 85% reduction whilst the 72 h LPS pre-exposure led to 32% reduction suggesting that inhibition of NF-κB activity in HI with sub-acute infection has limited efficiency. Plasminogen activator induction, in 72 h LPS pre-exposure, was preserved even after Tat-NBD administration highlighting the need for multi-faceted therapeutics in LPS-sensitised HI which will address the divergent pathological mechanisms underlying HI injury combined with sub-acute infection. Treatment with Tat-NBD had no therapeutic effect in pure-HI which further reinforces NF-κB as an integral contributor to LPS-sensitised HI brain damage.

### FTY720 (Fingolimod) – Sphingosine-1-Phosphate Receptor Agonist

FTY720 is a sphingosine 1-phosphate (S1P) receptor modulator approved for use in clinical care to treat multiple sclerosis (Brinkmann et al., 2010). Through agonistic interactions with lymphocytic S1P receptors, FTY720 causes internalisation and degradation of these receptors, thereby preventing the exit of lymphocytes, particularly TH17-lymphocytes, from the lymph nodes (Brinkmann et al., 2000, 2002; Mandala et al., 2002; Pham et al., 2010). Elevated levels of pro-inflammatory cytokines in LPS-sensitised HI, including IL-6 and IL-1β, are crucial for TH17-lymphocyte differentiation (Bettelli et al., 2006; Ghoreschi et al., 2010). Peripartum infection in neonates leads to increased TH17 circulation compared to other CD4 positive T cell subtypes. This bias toward TH17 cell differentiation is inversely correlated with age; the more preterm the infant the greater the tendency to generate TH17 cells (Black et al., 2012). Yang et al. (2014) suggested that systemic administration of FTY720 attenuates brain damage and behavioural deficits in P7 LPS-sensitised HI Wistar rat pups. FTY720 treatment reduced IL-17A-positive lymphocytes, lowered the levels of pro-inflammatory cytokines, attenuated BBB damage and protected CNS white matter and motor development. Moreover, FTY720 treatment had no effect in HI alone, emphasising the critical contribution of early-stage TH17 cells to neuroinflammation in LPS-sensitised HI (Yang et al., 2014).

### Vancomycin – Gram-Positive Bacterial Infection

Many cases of neonatal infections are due to Gram-positive bacterial sepsis. Although TH is neuroprotective in this instance, there is a need for supplementary therapies addressing the Gram-positive bacterial sepsis (Dong and Speer, 2015). Vancomycin downregulates LPS-induced TNF-α production (Siedlar et al., 1997) whilst upregulating anti-inflammatory (IL-10) cytokine activation (Ziegeler et al., 2006). In a preterm P4 mouse HI model pre-exposed to *Staphylococcus epidermis*, a bacterium commonly causing Gram-positive sepsis, the antibiotic vancomycin was neuroprotective (Lai et al., 2019). At 14 h post-injury it reduced bacterial load in the spleen, decreased caspase-3 activity and pro-inflammatory cytokine levels, and lowered white and grey matter loss assessed through immunohistochemical analysis. As well as being a promising candidate for Gram-positive bacterial sepsis, the anti-inflammatory properties of vancomycin make it a potential therapeutic option for Gram-negative bacterial sepsis.

### Properdin

The complement system is an important component of innate and acquired immunity with three pathways of activation: classical, lectin, and alternative pathways (Lesher et al., 2013). Properdin, a plasma glycoprotein, is a positive regulator of the complement system, released in the presence of pro-inflammatory cytokines (Lesher et al., 2013). It stabilises the alternative pathway convertases (C3bBb) through direct binding to C3b or through interactions with specific surfaces.

Data from patients with neonatal HI suggest decreased levels of C3 (Grether and Nelson, 1997) and an increase in C3a and C5a after foetal acidosis (Sonntag et al., 1998). Given the role of properdin in complement activation, the effects of global properdin deletion on LPS-sensitised HI injury as well as on HI injury alone, was investigated in a Rice–Vannucci model of neonatal HI (Sisa et al., 2019a). Global properdin deletion in P7 mice reduced brain damage in both HI alone and LPS-sensitised HI at 48 h post insult. In the model of HI-alone a reduction of 20–38% in cell death was observed in the pyriform cortex, hippocampus, striatum, and thalamus, as well as a 21–76% reduction in microglial activation. Global properdin deletion in LPS-sensitised HI injury reduced cell death (50–76%), tissue volume loss (13–66%), and microglial activation (31–66%). In both injury profiles global properdin deletion did not affect astroglial activation, suggesting that properdin is critical for the impaired microglial pro-inflammatory response in HI. These observations strongly associate properdin and complement activation with HI alone and LPS-sensitised HI injury highlighting its importance as a therapeutic target.

### Glucocorticoids

Glucocorticoids are steroids secreted by the adrenal gland in response to stressful stimuli. They have anti-inflammatory and immunosuppressive properties (De Bosscher et al., 2000). Glucocorticoids mediate inflammation through repressing pro-inflammatory cytokines such as TNF-α and IL-1β, and increasing anti-inflammatory cytokine expression as well as inhibiting NF-κB (Almawi and Melemedjian, 2002). Previously, it has been shown that dexamethasone decreased glucocorticoid receptor expression in neonatal HI injury in rats (Gonzalez-Rodriguez et al., 2014). Harding et al. (2017) investigated dexamethasone administration in HI alone, as well as hydrocortisone in LPS-sensitised HI injury in P7 rats. ICV dexamethasone administration after HI alone decreased overall brain infarction. However, prolonged dexamethasone administration increased cell death (Whitelaw and Thoresen, 2000). Therefore, due to fewer side effects with prolonged usage, hydrocortisone was investigated instead of dexamethasone (Feng et al., 2015). Hydrocortisone administration both intranasally and ICV, decreased infarction size after HI insult (Harding et al., 2017). Moreover, intranasal hydrocortisone administration post LPS-sensitised HI significantly decreased infarction size (Harding et al., 2017). Higher hydrocortisone doses decreased this effect which can be attributed to mediation of excitotoxic injury. Together, these results indicate a therapeutic role for glucocorticoids in both HI injury alone and LPS-sensitised HI.

### *N*-Acetylcysteine

*N*-acetylcysteine (NAC) is a free radical scavenger with antioxidant, anti-apoptotic (Ferrari et al., 1995), and anti-inflammatory (Louwerse et al., 1995) properties, and supplies cysteine which is critical for glutathione synthesis (Ghersi-Egea et al., 2006). It may also regulate glutamate levels through interaction with the cysteine/glutamate antiporter, thereby reducing neuronal glutamate release (Bridges et al., 2012). NAC reduced amniotic fluid and placental cytokine responses to LPS infection (Beloosesky et al., 2006) and stabilised oxidative balance (Xu et al., 2005). Due to the various therapeutic properties of NAC and its ability to cross the BBB (Farr et al., 2003) as well as its safe use in pregnancy (Beloosesky et al., 2006), NAC has therapeutic potential in LPS-sensitised HI brain injury. Wang et al. (2007b) investigated multiple NAC doses in P8 rats with LPS-sensitised HI injury (Xu et al., 2005). NAC (200 mg/kg) reduced infarct volume loss by 78.3% when administered both pre- and post-LPS-sensitised HI induction. Moreover, NAC treatment immediately after HI (0 h) led to greater reduction in brain injury (41%) compared with melatonin (Xu et al., 2005). Furthermore, NAC treatment reduced white matter injury, microglial activation, and redox signalling molecules, as well as nitrotyrosine and isoprostane production. Additionally, NAC treatment increased endogenous antioxidant molecules such as glutathione and thioredoxin-2 and suppressed caspase-3, calpain, and caspase-1 activation. Thus, a therapeutic role for NAC is feasible in LPS-sensitised HI injury.

### Downregulation of MicroRNA-21

MicroRNAs are small non-coding ribonucleic acid molecules implicated in various physiologic processes (He and Hannon, 2004). They are posttranslational regulators and act by binding to complementary sequences in mRNA, thereby suppressing or degrading target mRNA transcripts. MicroRNAs have been implicated in cellular growth, inducing proliferation, differentiation, suppressing apoptosis, and in regulation of inflammation (Montalban et al., 2014; Zhou et al., 2018). Given these properties, microRNAs have potential as therapeutic targets in LPS-sensitised HI injury. Zhou et al. (2018) investigated microRNA-21 (miR-21) downregulation in a P3 rat model of LPS-sensitised HI injury; where the animals were treated with antagomir-21 from the 2nd to 28th day post injury. miR-21 downregulation improved spatial learning and memory assessed through Morris water maze test. Moreover, miR-21 inhibition resulted in less vacuolar degeneration, better neuronal arrangement in the hippocampus, less neuronal oedema, and cell death compared to non-inhibited controls following LPS-sensitised HI injury. Therefore, mircroRNAs show therapeutic potential for infection-sensitised HI injury.

### PTEN-Induced Putative Kinase 1

PTEN-induced putative kinase 1 (PINK1) is a mitochondrial serine/threonine kinase well known for its role in Parkinson’s disease pathogenesis with PINK1 mutation leading to mitochondrial dysfunction and thereby neurodegeneration (Alexander, 2004). PINK1 has a critical role in mitochondrial quality control through identification and targetting of damaged mitochondria for degradation mediated *via* autophagy (Burchell et al., 2010). Some studies suggest a potential role for PINK1 in neuronal survival following HI injury (Chen et al., 2015; Li and Hu, 2015), as well as participation in cell proliferation through reprogramming of glucose metabolism (Requejo-Aguilar et al., 2014). Zhu et al. (2016) investigated the deletion of PINK1 in LPS-sensitised HI injury in P3 mice. Knockout of PINK1 in LPS-sensitised HI brain injury attenuated brain infarct volume at 24 and 72 h post insult. Additionally, at 24 h post insult PINK1-knockout animals had reduced levels of TUNEL positive cell death. PINK1-deletion increased α-Synuclein (α-Syn) expression, a downstream effector of PINK1 thought to suppress cell death (Bornhorst et al., 2014). Interestingly, inhibition of α-Syn with small interfering RNA reversed the neuroprotective effect observed in PINK1-knockout mice as brain infarct size and cell death increased (Zhu et al., 2016). Thus, PINK1 shows potential as a novel therapeutic target in LPS-sensitised HI injury.

A summary of the current neuroprotective agents for neonatal LPS-sensitised HI brain injury used in pre-clinical studies is shown in Table 2.

**TABLE 2:** Summary of neuroprotective agents for infection-sensitised neonatal brain injury.

Agent	Preclinical evidence
Histone deacetylase inhibitor (HDACi) trichostatin A (TSA)	Reduces white and grey matter injury, and cell death improves inflammatory profile and long-term learning in the mouse (Liu et al., 1989; Brogdon et al., 2007; Suh et al., 2010; Yakovlev et al., 2010; Fleiss et al., 2012).
Plasminogen activator inhibitor-1 (PAI-1 – CPAI)	Decreases brain damage, BBB damage, and inflammation *via* reduction of microglia activation and modulation of anti-inflammatory pathways in the rat (Zhang et al., 2007; Yang et al., 2009).
Cell-penetrating anti-NF-κB peptides (Tat-NBD)	Downregulates microglial activation and NfkB in the rat (May et al., 2000; Pizzi et al., 2009).
FTY720 (fingolimod) – sphingosine-1-phosphate receptor agonist	Reduction in IL-17A-positive lymphocytes, lower levels of pro-inflammatory cytokines, attenuated BBB damage, and protected brain white matter and motor development.
Vancomycin – Gram-positive bacterial infection	Downregulate LPS-induced TNF-α production (Siedlar et al., 1997) whilst upregulating anti-inflammatory, IL-10, cytokine production (Ziegeler et al., 2006).
Properdin	Reduces cell death, microglial activation (Sisa et al., 2019a).
Glucocorticoids	Prolonged administration of dexamethasone has been implicated in increased cell death (Whitelaw and Thoresen, 2000). Hydrocortisone administered both decreased infarction size (Harding et al., 2017).
*N*-acetylcysteine	Decreases acute brain edoema and sub-acute brain atrophy in a rodent model (Xu et al., 2005).
Downregulation of microRNA-21 (miR-21)	Increases caspase activity and lipid peroxidation in a rodent model injury (Wang et al., 2007b).
PTEN-induced putative kinase 1 (PINK1)	Attenuated brain infarct volume 24 and 72 h post insult, reduced cell death (Zhu et al., 2016).

## Conclusion

Basic science, translational, and clinical research of HIE have significantly expanded over the last two decades. Despite the advances in neonatal clinical care, the worldwide burden of HIE is substantial. TH is standard treatment for HIE, however, its application and efficacy are quite limited. Moreover, TH is not beneficial in infection-sensitised HI cases. Therefore, there is an unmet need for the development of new treatments to both complement and increase the efficacy, or to replace TH. The investigations of neuroprotective drugs and therapies for term and preterm HI neonates has significantly increased. Having in mind the pathology of HI, most approaches for both HI alone and infection-sensitised HI target inflammation, oxidative stress, and tissue loss in the short and long term, and aim to improve behavioural outcomes. Many promising agents such as resveratrol, cannabinoids, curcumin, and melatonin have been used in pre-clinical studies for both HI alone and infection-sensitised HI (Tables 1, 2). However, the ones with highest likelihood for success and closest to clinical implementation for HI alone include EPO for term and preterm HIE, and magnesium for antenatal prevention of preterm HIE. In the case of infection-sensitised HI the scenario is even more complicated, having in mind that Gram-positive and Gram-negative bacterial infections require different approach, which makes pre-clinical HI studies even more complex. In conclusion, given the enormous global socio-economic burden of the consequences from HIE, the search for therapies to prevent or treat the disease needs to continue and access to neuroprotective strategies for HIE in low resource settings needs to be improved.

## Author Contributions

KT and CS: collection of the literature, and writing and editing of the manuscript. AI and KD: collection of the literature and writing of the manuscript. MH: design, writing, and editing of the manuscript. All authors contributed to the article and approved the submitted version.

## Conflict of Interest

The authors declare that the research was conducted in the absence of any commercial or financial relationships that could be construed as a potential conflict of interest.

## Publisher’s Note

All claims expressed in this article are solely those of the authors and do not necessarily represent those of their affiliated organizations, or those of the publisher, the editors and the reviewers. Any product that may be evaluated in this article, or claim that may be made by its manufacturer, is not guaranteed or endorsed by the publisher.
